# Endoplasmic reticulum turnover: ER-phagy and other flavors in selective and non-selective ER clearance

**DOI:** 10.12688/f1000research.13968.1

**Published:** 2018-04-13

**Authors:** Ilaria Fregno, Maurizio Molinari

**Affiliations:** 1 Università della Svizzera italiana, Via G. Buffi, CH-6900 Lugano, Switzerland; 2 Institute for Research in Biomedicine, Via V. Vela 6, CH-6500 Bellinzona, Switzerland; 3 Department of Biology, Swiss Federal Institute of Technology, Wolfgang-Pauli-Strasse 27, CH-8093 Zurich, Switzerland; 4 École Polytechnique Fédérale de Lausanne, School of Life Sciences, EPFL Station 19, CH-1015 Lausanne, Switzerland

**Keywords:** Autophagy and macroautophagy, ER turnover, ER-phagy and recovER-phagy receptors, ER stress, LC3-interacting region (LIR), live bacteria, nutrient deprivation, proteostasis

## Abstract

The endoplasmic reticulum (ER) is a highly dynamic organelle in eukaryotic cells. It is deputed to lipid and protein biosynthesis, calcium storage, and the detoxification of various exogenous and endogenous harmful compounds. ER activity and size must be adapted rapidly to environmental and developmental conditions or biosynthetic demand. This is achieved on induction of thoroughly studied transcriptional/translational programs defined as “unfolded protein responses” that increase the ER volume and the expression of ER-resident proteins regulating the numerous ER functions. Less understood are the lysosomal catabolic processes that maintain ER size at steady state, that prevent excessive ER expansion during ER stresses, or that ensure return to physiologic ER size during recovery from ER stresses. These catabolic processes may also be activated to remove ER subdomains where proteasome-resistant misfolded proteins or damaged lipids have been segregated. Insights into these catabolic mechanisms have only recently emerged with the identification of so-called ER-phagy receptors, which label specific ER subdomains for selective lysosomal delivery for clearance. Here, in eight chapters and one addendum, we comment on recent advances in ER turnover pathways induced by ER stress, nutrient deprivation, misfolded proteins, and live bacteria. We highlight the role of yeast (Atg39 and Atg40) and mammalian (FAM134B, SEC62, RTN3, and CCPG1) ER-phagy receptors and of autophagy genes in selective and non-selective catabolic processes that regulate cellular proteostasis by controlling ER size, turnover, and function.

## The history behind regulated endoplasmic reticulum turnover

The volume (and the activity) of the endoplasmic reticulum (ER) can be expanded on demand. Activation of quiescent B cells into antibody-producing plasma cells is a textbook example of the daunting expansion of this biosynthetic organelle to accommodate and assist the maturation of a flood of cargo entering the secretory pathway (in this specific case, thousands of immunoglobulin chains produced per second) ^1^. In general terms, ER expansion is one of the many cellular responses to ER stresses, which collectively are defined as unfolded protein responses (UPR). Logically, recovery of pre-stress homeostasis upon ER stress resolution or cessation of drug (e.g. phenobarbital) treatments requires dismantling of the excess ER and degradation of the ER chaperones produced during the stress phase ^2^. It is in these contexts that first indications of catabolic control of ER size emerged ^3– 5^. However, hints of lysosomal-regulated ER turnover can be found in earlier literature (for example, in 6). Selective ER turnover is a constitutive process that maintains ER size in Eukarya. It can be enhanced or induced on microtubule de-polymerization ^7^ and, as described below, during ER stress, during recovery from acute ER stress, on ER overload with membrane or proteasome-resistant misfolded proteins, under nutrient deprivation, or on pathogen attack. It relies on intricate mechanisms that we are just starting to understand.

## Yeast flavors of ER-phagy

### Chapter one: the origin of a name. ER-phagy as ER stress-induced microautophagy of the ER

The term ER-phagy is a contraction of the words “ER” and “macroautophagy”. It was coined by the group of Peter Walter to define the process of selective ER delivery (not of selective ER clearance) in the vacuole of yeast cells exposed to perturbation of the redox homeostasis to induce a UPR ^8^. Initially, ER-phagy was described as a conventional macroautophagic pathway with autophagy genes alleviating cell growth defects under ER stress conditions and regulating the engulfment of ER fragments within double-membrane autophagosomes that eventually fuse with vacuoles to clear their content ^8, 9^. None of these original findings survived the test of time. In fact, further analyses by the same group revealed that neither autophagy genes nor autophagosomes are actually involved in the vacuolar delivery of excess ER upon perturbation of redox homeostasis or play a role in the survival of yeast cells challenged with the reducing agent dithiothreitol (DTT). Moreover, it turns out that ER delivery to the vacuole under these experimental conditions does not result in ER clearance ^10^ ( Table 1). Rather, on chemical UPR induction, excess ER membranes are delivered within the vacuole by poorly understood micropinocytosis events. Importantly, because DTT inactivates vacuolar activity, the ER delivered into the vacuole under these experimental conditions accumulates in the vacuolar lumen as ER whorls ^10^. The pathways and the yeast genes regulating ER fragmentation, selective delivery of ER whorls within vacuoles (is it controlled by ER-phagy receptors?), and capture of ER by microautophagic invagination of the vacuolar membrane remain to be identified.

**Table 1.** Yeast ER-phagy types and their requirements.

ER-phagy type (yeast)	Receptor	Gene products required	Gene products dispensable
Dithiothreitol-induced microER-phagy ^8– 10^	Unknown	Unknown	Atg1, Atg7, Atg8, Atg16, Ego1, Ego3, Vtc4, Nvj1, Pep4, Vps4, Vps23
Membrane protein-induced macroER- phagy ^11^	Unknown	Atg1, Atg2, Atg8, Atg9, Atg11, Ypt1, Ypt51	Atg17, Atg18, Atg19, Atg32, Atg36
Nutrient deprivation-induced/rapamycin- induced ER-phagy ^15^	Atg39 or Atg40	Atg1, Atg8, Atg11, Atg17, Ypt7, Pep4	Unknown

### Chapter two: membrane protein-induced macroautophagy of the ER

Overexpression of integral membrane proteins is yet another manner to activate/enhance delivery of ER to the yeast vacuole ^11, 12^. This process is highly selective because endogenous markers of the ER membrane (Sec61 and Hmg1) but not intrinsic or peripheral proteins of ER exit sites (Sec12 and Sec13) or the luminal ER protein Kar2p are delivered to the vacuole with the exogenous integral protein(s) to be cleared from cells ^11^. Membrane protein-induced delivery of ER material to the yeast vacuole is under the control of Ypt1 (the orthologue of mammalian RAB1) to generate ER-to-autophagy membrane structures, of Ypt51 (Vps2) to promote fusion of ER membrane proteins-containing structures with the vacuole, and of select macroautophagy gene products (Atg1, Atg2, Atg8, Atg9, and Atg11 but not Atg17, Atg18, Atg19, Atg32, and Atg36 ^11^; for a description of the function of the various autophagy gene products mentioned in this commentary, please refer to 13, 14) ( Table 1). The role of Atg39 and Atg40 as ER-phagy receptors in nutrient deprivation-induced yeast ER-phagy was published subsequently (chapter three in this commentary and 15). To our knowledge, a possible involvement of Atg39 or Atg40 (or both) in membrane protein-induced selective macroautophagy remains to be investigated.

### Chapter three: nutrient deprivation-induced, receptor (Atg39 and Atg40)-mediated macroautophagy of the ER (and of the nuclear envelope)

**Figure 1.**
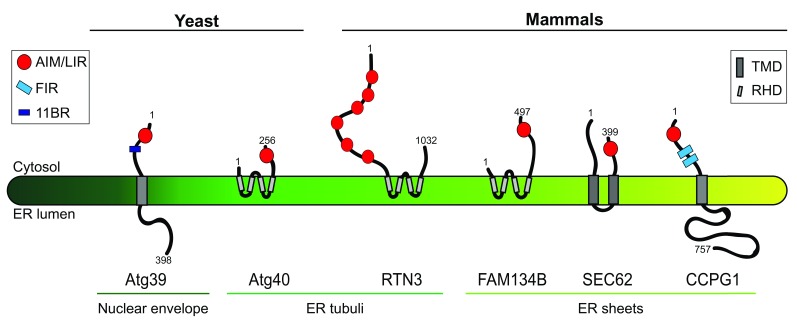
Yeast and mammalian ER-phagy receptors. The figure shows the topology, orientation, and subcompartmental localization of the two yeast ER-phagy receptors Atg39 and Atg40 and of the four mammalian ER-phagy receptors RTN3, CCPG1, FAM134B, and SEC62. Numbers indicate amino acid residues and refer to the human version of the proteins for the mammalian ER-phagy receptors. 11BR, Atg11-binding region; AIM, Atg8-interacting motif; ER, endoplasmic reticulum; FIR, FIP200-interacting region; LIR, LC3-interacting region; RHD, reticulon-homology domain; TMD, transmembrane domain.

Nutrient deprivation and rapamycin are conventional activators of macroautophagy ^16^. Like cell exposure to DTT ^10^, these experimental conditions enhance the delivery of ER fragments within the yeast vacuole. However, as crucial differences (compare with chapter one), vacuolar delivery eventually results in ER clearance ^15, 17^ and relies on macroautophagy-like programs, where receptor-mediated engulfment of ER fragments into double-membrane autophagosomes that eventually fuse with lysosomes does indeed occur ^15, 17, 18^. Atg39 spans the perinuclear ER membrane and decorates it for capture by autophagosomes and destruction. Instead, Atg40 is in the peripheral ER membrane, where it co-localizes with Rtn1, a marker of ER tubules and sheet edges, but not with the ER sheet marker Sec63 and regulates the turnover of highly curved ER subdomains ( Figure 1). Atg39 and Atg40 contain an Atg8-interacting motif (AIM) that bridges the ER membrane with the phagophore membrane via Atg8 association. Moreover, Atg40 (and possibly Atg39) contains an Atg11-binding region (11BR, Figure 1) and harbors two transmembrane domains that have some similarities with reticulon domains displayed by some mammalian ER-phagy receptors ( 15, see below). These domains supposedly curve the ER membrane to facilitate the ER fragmentation required for engulfment by autophagosomes. So far, it has been established that nutrient deprivation-induced, Atg39- and Atg40-mediated macroautophagy of the yeast nuclear envelope and ER requires Atg1 (ULK1 in mammals), Atg8 (LC3), Atg11 and Atg17, Ypt7 (RAB7, a small GTPase required for autophagosome-vacuole fusion), and Pep4 (a vacuolar peptidase) ^15^ ( Table 1).

## Mammalian flavors of ER-phagy

### Chapter four: nutrient deprivation-induced, receptor (FAM134B or RTN3)-mediated macroautophagy of the ER

As in yeast cells, starvation activates receptor-mediated engulfment of mammalian ER fragments into double-membrane autophagosomes that eventually fuse with lysosomes to clear their cargo ^19, 20^. The reticulon family members FAM134B and RTN3 reside to the edges of ER sheets and ER tubuli, respectively ( Figure 1). FAM134B was identified in a yeast two-hybrid screen as a LC3/GABARAP interactor ^19^. RTN3 was found to re-localize from ER tubuli to LC3-decorated structures on cell starvation ^20^. Upon oligomerization, FAM134B and RTN3 fragment ER sheets and tubuli, respectively, and mediate the capture of these ER subdomains by autophagosomes via their LC3-interacting regions (LIRs). As such, different ER-phagy receptors regulate the turnover of distinct populations of ER-resident proteins, according to the subcompartmental distribution of the latter in sheets (for example, CLIMP63 and RTN4B) or in tubuli (RTN1, RTN3, REEP5, and CNX). Since inactivation of ER-phagy receptors causes ER expansion, ER turnover is a constitutive process that also determines ER size at steady state. So far, it has been established that nutrient deprivation-induced, FAM134B- and RTN3-mediated ER-phagy requires BCN1, ATG5, ATG7, LC3, and FIP200 ^19, 20^ ( Table 2).

**Table 2.** Mammalian ER-phagy types and their requirements.

ER-phagy type (mammalian)	Receptor	Gene products required	Gene products dispensable
Nutrient deprivation-induced ER-phagy ^19, 20^	FAM134B	BCN1, FIP200, ATG5, LC3	RTN3
	RTN3	FIP200, ATG5, ATG7, LC3	FAM134B
Nutrient deprivation-induced/ ER stress-induced ER-phagy ^23^	CCPG1	FIP200, ATG5, LC3	Unknown
RecovER-phagy ^2^	SEC62	ATG5, ATG7, LC3	Unknown
Microbial-induced ER-phagy ^24^	Unknown	BCN1, FIP200, ATG7, ATG14, ATG16L1	FAM134B

### Chapter five: receptor (SEC62)-mediated clearance of excess ER on resolution of acute ER stresses (recovER-phagy)

Pathologic (for example, expression of mutant polypeptides) or physiologic (for example, B- to plasma-cell differentiation) biosynthetic challenges may induce ER stresses that increase the ER volume to almost entirely occupy the space delimited by the plasma membrane ( http://www.drjastrow.de/WAI/EM/EMRERE.html). This can be recapitulated by challenging cells with chemical compounds or by therapeutic interventions that perturb ER homeostasis ^21^. Alleviation of the stress obtained on interruption of the challenge allows cells to re-establish pre-stress homeostatic conditions by lysosomal clearance of the excess ER membranes and proteins produced during the stress phase ^2– 5^. This catabolic process, defined as recovER-phagy (for ER-phagy-mediated recovery from ER stresses ^2^), is characterized by selective clearance of ER subdomains displaying the LC3-binding protein and (recov)ER-phagy receptor SEC62 at the limiting membrane ^2^ ( Figure 1). SEC62 plays a well-characterized role as a component of the protein import machinery into the ER ^22^. Bioinformatics analyses revealed the presence of a LIR in the cytosolic C-terminus of SEC62, which is conserved in higher Eukarya but not in the yeast orthologue. LC3 binding to SEC62 has been mapped by docking and molecular dynamics simulations and by solution nuclear magnetic resonance spectroscopy. It has been confirmed by surface plasmon resonance and peptide array and eventually in the living cell, where it proved to be required for the delivery of ER fragments within LAMP1-positive degradative organelles ^2^. Proteomic analyses revealed that the ER subdomains cleared by recovER-phagy do contain several ER-resident molecular chaperones and enzymes (for example, all members of the protein disulfide isomerase superfamily with the relevant exception of ERp44, which resides not in the ER but in the intermediate compartment ^25^) but are devoid of other ER-resident proteins such as most ERAD regulators ^2^. This reveals a spatial separation of ER functions in ER subdomains. So far, it has been established that SEC62-mediated recovER-phagy requires ATG5, ATG7, and LC3 ( Table 2).

### Chapter six: ER stress-induced receptor (CCPG1)-mediated macroautophagy of the ER

CCPG1 is the new entry in the ER-phagy receptors clan. It has been identified as an ER stress-induced interactor of GABARAP and LC3 ^23, 26^. It is a single-pass, type II ER protein that associates directly with FIP200 and indirectly with other components of the ULK complex involved in autophagosome biogenesis (that is, ULK1, ATG13, and ATG101) ^23^ ( Figure 1). Ectopic expression of CCPG1 reduces the size of the peripheral ER and the cellular content of the peripheral/tubular ER protein RTN3. Likewise, induction of endogenous CCPG1 in cells challenged with the chemical ER stress inducer DTT activates lysosomal depletion of peripheral ER. DTT induction of yeast ER-phagy results in microautophagy-like vacuolar capture of the ER, which does not require intervention of autophagy gene products and does not lead to ER degradation (Chapter one ^10^). Thus, the mechanism of ER size control in cells experiencing chemically induced ER stresses is poorly conserved, if at all. Of note, FAM134B deficiency is linked to human sensory neuropathy ^19^, whereas studies in mice reveal that CCPG1 deficiency causes injury of exocrine pancreas cells and of chief cells of the gastric epithelium ^23^. These differences reveal poorly understood tissue-specific functions of the two ER-phagy receptors. CCPG1-mediated ER-phagy requires ATG5 and FIP200 ( Table 2). 

### Chapter seven: misfolded protein-induced macroautophagy of the ER (ER quality control autophagy)

Most misfolded polypeptides produced in the ER are translocated across the ER membrane and are degraded by cytosolic 26S proteasomes ^27^. However, the polymerogenic E342K (ATZ) variant of the secretory protein alpha1 antitrypsin ^28– 31^, the E90K mutant of the gonadotropin-releasing hormone receptor (GnRHR) ^32^, aggregated β subunits of thyrotrophic hormone ^33^, procollagen ^34, 35^, and dysferlin ^36^ are a few paradigmatic examples of proteasome-resistant proteins generated in the ER and degraded, at least in part, by the lysosomal system. For ATZ, the involvement of classic autophagy regulators such as ATG5, ATG7, and LC3 has been shown ^31^. However, for this and other proteasome-resistant misfolded polypeptides such as GnRHR and β subunits of thyrotrophic hormone, the intervention of classic (mTOR-dependent) macroautophagy pathways in clearance is questionable. In fact, macroautophagy inducers such as rapamycin and starvation do not enhance their lysosomal disposal ^30, 32, 37, 38^, which seems to actually require vesicular traffic from the ER to lysosomal degradative compartments ^33, 39^. In these cases, the autophagy genes involved in these catabolic processes might intervene in an ill-defined, unconventional manner. Even if/when conventional macroautophagy determines the clearance of proteasome-resistant misfolded proteins generated in the ER lumen, it remains unclear whether these are dislocated across the ER membrane and subsequently are captured by autophagosomes or whether autophagosomes capture ER fragments containing aberrant protein aggregates to then fuse to lysosomes ^31^. In the latter case, it remains to be established how the autophagic machinery operating in the cytosol can detect the accumulation in the ER lumen of proteasome-resistant species to be removed from cells (see “Final remarks”). All in all, the involvement of peculiar pathways (autophagy-like or vesicular transport or both), of ER-resident sensors that transmit the information from the ER lumen into the cytosol to activate autophagy, and of ER-phagy receptors to promote the catabolic process that removes the ER subdomain from cells seems to be an educated guess.

### Chapter eight: microbial-induced ER-phagy

Cyclic-di-adenosine monophosphate is a signature of live Gram-positive bacteria, which is detected by phagocytes to trigger a protective ER stress response consisting of ER expansion and chaperone gene upregulation. Subsequently, ER stress-induced PERK phosphorylation correlates with mTORC1 inactivation, thus inducing macroautophagy, which is instrumental for cell survival ^24^. Isolation of autophagosomes from cells challenged with live bacteria revealed the presence of several ER markers and the absence of markers of ERGIC, Golgi or lysosome, showing that live bacteria induce selective ER-phagy. A role for FAM134B as a receptor for microbial-induced ER-phagy has been excluded. The involvement of other known ER-phagy receptors has not been tested ^24^. So far, it has been established that microbial-induced ER-phagy requires ATG7, ATG14, ATG16L1, BCN1, and FIP200 ( Table 2).

## Promiscuous autophagic receptors in ER-phagy, an addendum: the case of p62 and BNIP3

As originally reported for other drugs ^3– 5^, withdrawal of hepatic mitogens activates the removal of excess ER from liver cells, which is ensured by macroautophagic processes. In these *in vivo* experiments, ER turnover required ATG5 and the general autophagy receptor Sequestosome1/p62 ^40^. In contrast to conventional ER-phagy receptors, which are located in the ER membrane ( Figure 1), p62 is a cytosolic protein that links ubiquitylated proteins to be degraded to the autophagic machinery via LC3 interaction. It is therefore likely that p62 regulates the clearance of ER regions displaying heavily ubiquitylated proteins at the cytosolic face of the membrane. A second intriguing case of promiscuous receptors involved in ER turnover is that of BNIP3, which is anchored primarily in the outer mitochondrial membrane via a C-terminal transmembrane domain ^41^. The BNIP3 homologue NIX/BNIP3L preferentially binds GABARAP ^42^ and regulates the removal of damaged mitochondria ^43^. BNIP3 selectively removes damaged mitochondria on association with LC3B ^44^. The finding that a subfraction of cellular BNIP3 is also found in the ER membrane led to the postulation that this protein could play a role as an ER-phagy receptor ^44^. This was experimentally demonstrated only on ectopic expression of a BNIP3 version modified for preferential delivery into the ER membrane ^44^.

## Final remarks

Autophagy was once considered a rather unselective pathway to deliver faulty material to lysosomes for clearance. Recent studies reveal the specificity and sophistication of autophagic programs and of programs relying on unconventional roles of autophagy genes ^45^. Organelles such as mitochondria, peroxisomes, nucleus, and ER can selectively be delivered to the lysosomal pathway for destruction if and when they display receptors at the surface that engage this intricate catabolic machinery ^46^. These receptors are constitutively active, for example, to control the size of the ER at steady state or in resting cells. They can be activated on demand to recover pre-stress ER size and content or in response to accumulation in specific ER subdomains of misfolded polypeptides that cannot be handled by the ubiquitin proteasome system. The study of ER-phagy actually reveals that not only organelles but also specific (functional) subdomains of an organelle, with their content, can be selected for destruction. The field is young and relies mostly on studies performed in cells exposed to exogenous stimuli such as nutrient deprivation or chemical stress that activate selective and non-selective ER-phagy and have uncontrolled pleiotropic consequences on many unrelated pathways ^47^. Intrinsic signals (that is, signals originating from the membrane or the lumen of confined ER subcompartments such as accumulation of proteasome-resistant polypeptides) are predicted to activate highly specific, receptor-controlled pathways relying on different autophagy, autophagy-like, or autophagy-independent lysosomal pathways. We also predict that studies on ER turnover will lead to the identification of ER sensors that, much like ER stress sensors, signal accumulation of proteasome-resistant misfolded proteins or other stressful situations that must be resolved by ER clearance. Analysis of the available literature already shows that ER-phagy comprises a series of mechanistically distinct processes that regulate the delivery of ER fragments or their luminal content (or both) within vacuoles/lysosomes. It is proposed, but in most cases not yet experimentally demonstrated, that these catabolic processes regulate ER turnover, ER size, and clearance of ER subdomains containing proteins and lipids that are faulty or present in excess. Intriguingly, under some pathologic conditions (for example, in some serpinopathies ^28^) or in a subset of patients (for example, 10% of the ATZ patients that show hepatotoxicity due to intracellular accumulation of ATZ polymers ^31^) or in response to severe chemically induced ER stresses ^8– 10^), the ER-derived material accumulates in autophagosomes or in degradative organelles attesting defective clearance. In other cases, accumulation of ER fragments in degradative organelles occurs only on inactivation of lysosomal hydrolases, rather hinting at a very efficient catabolic process operating to protect cell and organism viability. Current models show that ER fragments are captured by autophagosomes as normally happens for cytosolic material. However, other mechanisms of ER delivery to degradative organelles such as vesicular transport can be envisioned on the basis of available literature ^33, 39^. It will be of great interest to study the involvement of the several known autophagy genes in ER turnover to understand why treatment with conventional autophagy activators is beneficial for the clearance of cytosolic aggregates ^48^ and for some (for example, pro-collagen and dysferlin ^34– 36^) but apparently not for other (for example, ATZ and the E90K mutant of GnRHR) ^30, 32, 37, 38^ proteasome-resistant misfolded polypeptides generated in the ER lumen.
